# Mechanical Optimization of Concrete with Recycled PET Fibres Based on a Statistical-Experimental Study

**DOI:** 10.3390/ma14020240

**Published:** 2021-01-06

**Authors:** Alejandro Meza, Pablo Pujadas, Laura Montserrat Meza, Francesc Pardo-Bosch, Rubén D. López-Carreño

**Affiliations:** 1 Department of Mechanical Engineering, Tecnológico Nacional de México/IT Aguascalientes, Av. Adolfo López Mateos 1801, Aguascalientes 20256, Mexico; alejandro.meza@mail.ita.mx; 2 Department of Project and Construction Engineering, Universitat Politècnica de Catalunya (UPC-Barcelona Tech), Escola Tècnica Superior d’Enginyers Industrials de Barcelona (ETSEIB), Av. Diagonal, 647, 08028 Barcelona, Spain; 3 Department of Engineering, Tecnológico Nacional de México/IT Aguascalientes, Av. Adolfo López Mateos 1801, Aguascalientes 20256, Mexico; lauraa_mezaa@hotmail.com; 4 Department of Project and Construction Engineering, Universitat Politècnica de Catalunya (UPC-Barcelona Tech), Escola Superior d’Enginyeries Industrial, Aeroespacial i Audiovisual de Terrassa, Carrer de Colom, 15, 08222 Terrassa, Spain; francesc.pardo@upc.edu; 5 Independent Researcher, 08036 Barcelona, Spain; rubenlc@gmail.com

**Keywords:** recycled fibres, PET bottles, fibre reinforced concrete, optimization, statistical analysis

## Abstract

Discarded polyethylene terephthalate (PET) bottles have damaged our ecosystem. Problems of marine fauna conservation and land fertility have been related to the disposal of these materials. Recycled fibre is an opportunity to reduce the levels of waste in the world and increase the mechanical performance of the concrete. PET as concrete reinforcement has demonstrated ductility and post-cracking strength. However, its performance could be optimized. This study considers a statistical-experimental analysis to evaluate recycled PET fibre reinforced concrete with various fibre dose and aspect ratio. 120 samples were experimented under workability, compressive, flexural, and splitting tensile tests. The results pointed out that the fibre dose has more influence on the responses than its fibre aspect ratio, with statistical relation on the tensional toughness, equivalent flexural strength ratio, volumetric weight, and the number of fibres. Moreover, the fibre aspect ratio has a statistical impact on the tensional toughness. In general, the data indicates that the optimal recycled PET fibre reinforced concrete generates a superior performance than control samples, with an improvement similar to those reinforced with virgin fibres.

## 1. Introduction

Plastics are widely used throughout the world, contributing enormously to their industrial development, helping to raise the living standards in the world [1]. Plastics bring many societal benefits and offer future technological and medical advances. However, concerns about their usage and disposal are diverse [2]. Tons of plastic debris are discarded every year, generating pollution of lands, rivers, coasts, beaches, and oceans. Plastics have a significant adverse impact on marine life, onshore and offshore. According to the National Oceanographic and Atmospheric Administration, plastic debris kills an estimated 100,000 marine mammals annually, as well as millions of birds and fish [3]. Between 1950 and 2017, 6.4 billion metric tons of plastic waste were produced globally, of which 9% was recycled, 79% went into landfills, and 12% was incinerated [4]. Most plastics are non-biodegradable, and some could take anywhere from 100 to 1000 years to decompose in landfills [5]. The global waste plastic production per year has been reported in Europe as 288 million tons [6], in India as 18.9 million tons [7], in the USA as 31.75 million tons [8], in the Asian region as 6.7 million tons [9], in Korea as 130 thousand tons [9], and in the UK as 4.7 million tons [10].

Polymers are elements composited by a long chain of repeating molecules. Their advantages are low density, good strength-to-weight ratio, corrosion resistance, and low thermal conductivity. According to their molecular crosslinking and their properties, polymers are thermoplastics, thermosets, and elastomers. Polyethylene terephthalate (PET) is a thermoplastic polymer resin with a profitable strength-to-weight relation, high durability, low cost [6,7,8], and widely used in construction, transportation, packaging, and engineering applications [11]. In 2020, global PET production reached 70 million metric tons, taking more than 15% of the worldwide synthetic polymer production capacity [12]. Recycling PET wastes, and additional expenses are required for reprocessing. Thus, an effective and green solution is needed for polymers wasted [13] to balance the economic and environmental impacts on a long-term approach [14].

Cement-based materials are the most widely used construction materials mainly due to their high compressive strength as well as long service life, and low cost. However, those materials have the inherent disadvantages of low tensile strength and crack resistance. The addition of short, discrete fibres provides enhanced properties to this composite cement-based material [15,16]. In the hardened state, the bridging effect of the fibres increases the post-cracking residual strength of the material. Fibres modify the non-linear structural behaviour of concrete in tension, reducing the opening of cracks and counteracting their propagation [17,18]. The debonding and pull-out mechanisms of the fibres generate a considerable amount of dissipated energy, leading to the improvement of the concrete toughness [17,18]. This enhanced behaviour is influenced mainly by the number of fibres crossing a crack effectively, and the bond and strength properties of the reinforced elements used [18]. Plastic fibres attracted the attention of researchers as a reinforcement for concrete materials during the first half of the 1960s. These fibres are chemically inert and very stable in the alkaline concrete medium, and their use may be a cost-effective solution for elements with improved durability (e.g., an increased technical lifetime of structures and architectural applications). Among the different plastic macro-fibres used in fibre reinforced concrete (FRC), polyolefin-based fibres such as polypropylene (PP) are the most common [17,18,19].

Studies have proposed the use of the discarded material to reinforce the concrete and produce an FRC. Shaikh [20] studied the tensile and flexural behaviour of recycled polyethylene terephthalate fibre reinforced geopolymer composites, indicating that the composite had good mechanical strength. Borhan et al. [21] tested concrete reinforced with fibres from waste materials, pointed out that the FRC with polyvinyl waste elements affected the slump behaviour. Meza and Siddique [22] showed a considerable augment on the residual strength of the FRC with waste PET fibres under flexural load. Some researchers [23] studied concrete reinforced by plastic fibres based on local materials, indicating that the addition of 1.5% of PET fibres into concrete increases the compressive strength. Khalid et al. [24] tested FRC with PET fibre; the results indicated good splitting tensile strength. Lin et al. [25] analysed FRC with short PET fibres, and pointed out that the samples with recycled elements had mechanical properties comparable to high-performance fibres. Mastali [26] investigated the feasibility of using polymeric recycled fibres in concrete; the data indicated that fibres increased the impact resistance. Mansour and Ali [27] proposed the use of PET bottles to produce blocks for construction applications. Also, different statistical analyses have been carried out to analyse the effect of the reinforced parameters on steel fibre reinforced concrete (SFRC). Ayan et al. [28] used Taguchi’s and ANOVA methodologies to investigate the compressive behaviour of concrete reinforced with commercial steel fibres, considering different fibre volume fractions. Dvorkin et al. [29] proposed a method to search the optimal design of the concrete with steel fibres, considering the influence of the fibre content on the compressive and flexural strength. Bayramov et al. [30] studied the effects of aspect ratio and volume fraction of commercial steel fibres on the fracture behaviour of the concrete; the technique used was the response surface method. However, little or no research has been conducted on the effect of those parameters on recycled PET fibre reinforced concrete (R/PET-FRC).

Despite the remarkable advances that have been attained in the field of recycled PET fibre reinforced concrete (R/PET-FRC) in terms of its feasibility as a building material, the successful use of recycled PET fibre as an alternative to conventional fibre reinforcement still requires thorough research on the material behaviour and the influence of the addition of recycled fibres. Thus, the performance of the recycled fibres reinforced concrete must be studied thoroughly to understand their mechanical behaviour. To gain insight on this issue, a full detailed factorial statistical analysis on recycled PET fibre reinforced concrete is carried out in this manuscript. The results represent a meaningful contribution to the R/PET-FRC performance based on dosage and aspect ratio design optimization of the recycled PET fibres and provide an answer to the main questions hindering the extensive use of this material. The fibres are produced from PET bottles collected without any selection, increasing the use of discarded bottles for recycling.

## 2. Research Approach

**Figure 1:**
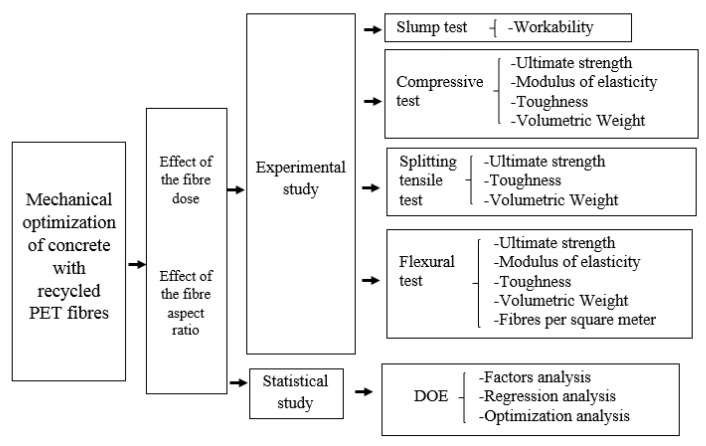
Tests considered in the experimental campaign.

The main objective of this study is to analyse the effect of the recycled PET elements on the performance of fibre-reinforced concrete samples, searching for the best relation of fibres dose and dimension to optimize the combined responses of workability, volumetric weight, compressive strength, flexural strength, and splitting tensile strength. The study is supported by a statistical method (full factorial study) to obtain conclusions. The results demonstrated that the recycled PET fibres generate change in the mechanical performance of the concrete, with an optimal solution on concrete samples with a high proportion of fibre dose. The information available in this paper provides the project engineer with the opportunity to apply this material more confidently. Therefore, this research contributes to the knowledge of R/PET-FRC, thus contributing also to spread its use. Figure 1 shows a relation between the tests performed on the experimental campaign and the parameters considered.

## 3. Materials and Methods

The experimental campaign is planned according to the design of experiments (DOE). DOE is a statistical method that studies the interaction between the factors, considering the difference between the responses when the levels are changed [28,31]. In this study, the factors were (1) the recycled fibre dose and (2) aspect ratio, with three references: a lower level of 2 kg/m^3^ and 50 mm/mm, an upper level of 10 kg/m^3^ and 100 mm/mm, and a central level 6 kg/m^3^ and 80 mm/mm. The tests considered sample replication and randomization. Sample replication allows estimating the experimental error and evaluates the difference between the responses and the random runs; this permits balancing the effect of external or uncontrollable conditions that could influence the results. Ayan et al. [28] recommend the DOE method to analyse the performance of concrete, due to the possibility to obtain statistical conclusions with a reduced number of experiments.

Full factorial DOE studies all the combinations between the factors and the levels. The relation 2 k gives the number of runs, where the k is the two factors considered (recycled PET fibre dose and aspect ratio). In this study, four statistical combinations are required, integrated by the relations 10-110, 10-50, 2-110, and 2-50 (in reference to dose in kg/m^3^ and aspect ratio in mm/mm, respectively); also, a central relation (samples 6-80) is used to know the behaviour between the limits. Moreover, the study considers control samples (of plain concrete, without fibres) to compare the performance of the recycled PET fibres reinforced concrete. All the sets of samples had five replications, with a total of 120 tests, which represents a relevant cross-wise comparison of 120 experimental tests with different recycled PET fibre reinforced concrete (R/PET-FRC).

### Materials, Mixing Procedure and Casting

**Figure 2:**
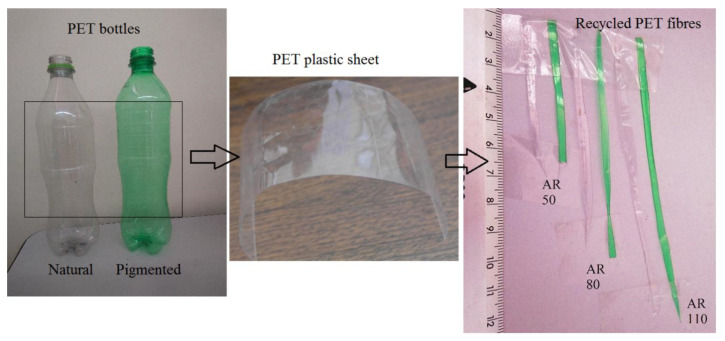
Procedure to generate recycled polyethylene terephthalate (PET) fibres (AR is the fibre aspect ratio).

Discarded PET bottles of carbonated drinks were collected to produce the recycled fibres. Although the objective was the collection without any selection, the trend found is natural and pigmented bottles of 0.6, 1.5, and 2 L. After the bottle collection, a plastic sheet was generated by removing the top and bottom bottle parts. Then the plastic sheet was cut with a paper cutter guillotine to produce the reinforced elements, with three longitudes (53.5, 85.6, and 117.8 mm). All the recycled fibres had a thickness of 0.3 mm and a width of 3 mm. The aspect ratio calculated according to the CNR [32] indicated an aspect ratio of 50, 80, and 110, respectively. Figure 2 shows the production procedure of recycled fibres, and Table 1 shows the mix proportion composition for the concrete samples.

**Figure 3:**
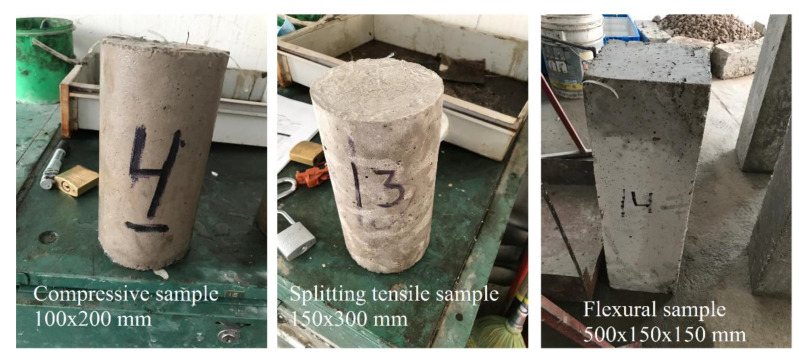
Compressive, flexural, and splitting tensile samples produced for each run.

The preparation of the concrete samples with recycled PET fibres started with the weighing and mixing of materials. The materials and proportion were ordinary Portland cement type I (383 kg/m^3^), two aggregates: natural sand (672 kg/m^3^), and medium gravel of 20 mm (1100 kg/m^3^). All the samples had the same water/cement ratio (0.6), which is similar to a previous study [21]. The density in g/m^3^ was 3.15, 2.50, and 2.68 for the cement, natural sand, and medium gravel, respectively. The mixing procedure consisted of stirring up manually the cement, aggregates, and fibres for three minutes, then the water was dispersed, and the material was mixed for another 3 min to get homogeneity. In total, 30 batches were generated, which comprise the five different combinations of recycled PET fibres and the control samples, all with five replicates. Each batch had enough materials for three specimens, corresponding to compressive, flexural, and splitting tensile tests (with dimensions of 100 × 20 mm, 150 × 150 × 500 mm, and 150 × 300 mm). The specimens were cast in steel moulds and cured for 28 days, according to ASTM C192 [33]. Figure 3 depicts three samples produced on each run.

## 4. Results and Discussion

### 4.1. Slump

**Figure 4:**
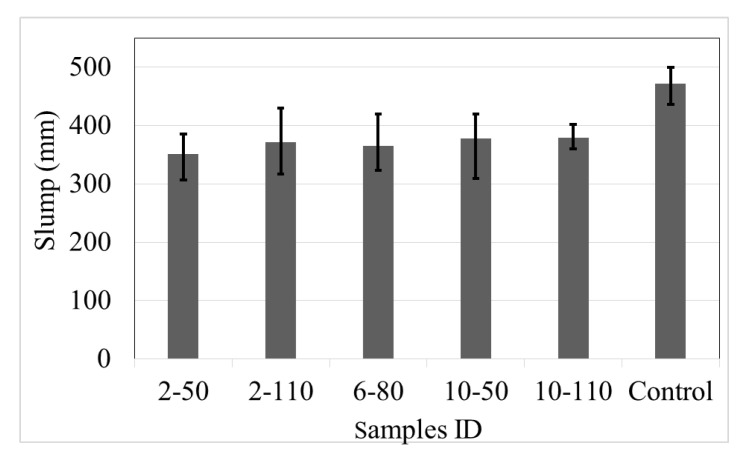
Slump results of control samples and concrete with recycled PET fibres.

The slump test, according to ASTM C995 [34], was used to evaluate the workability of the concrete mixes. This method, the inverse slump cone test, is recommended to assess the behaviour of fibre-reinforced concrete batches [35]. Figure 4 shows the average and limits results considering the five replicates in each set.

The results indicate that control samples had a slump value of 472 mm, which is 22% superior that the mean of batches with recycled PET fibres. Also, the incorporation of recycled PET fibres to the concrete augments the variation in the slump response. On the other hand, the effect of the fibre dose and aspect ratio on the workability is scarce, with a maximum difference of 7.6%. Highlighting that the batches with the highest recycled PET fibre dose and aspect ratio demonstrated the best slump, a phenomenon attributed to the low rigidity of the recycled PET fibres [36].

### 4.2. Compressive Behaviour

Concrete cylinder specimens of 100 × 200 mm (diameter and length, respectively) were tested according to ASTM C39 [37]. The deformation was measured with a plunger dial indicator located on the structure of the universal testing machine since the displacement of the load plates has a direct relation with the deformation of the sample. The dial indicator covered a measuring range from 0 to 25 mm with resolution of 0.25 µm and accuracy of ±0.25 µm.

**Figure 5:**
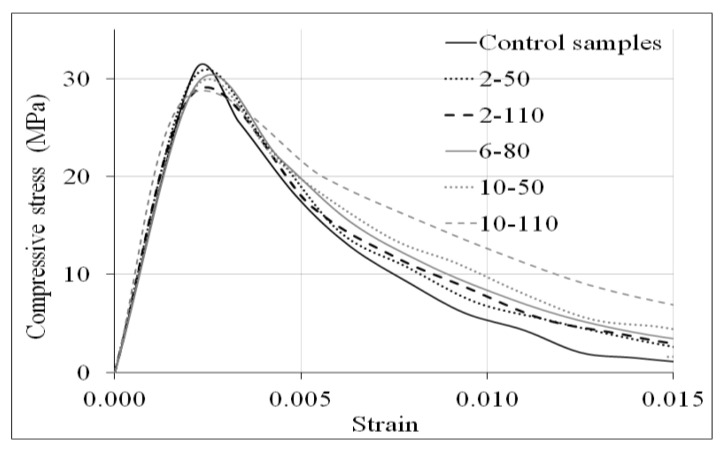
Compressive stress-strain curves of control samples and concrete samples with recycled PET fibres.

Figure 5 shows the compressive stress-strain curves of the control concrete and those with recycled PET fibres. For each combination of fibre dose and aspect ratio, a curve was represented with the average strengths of 5 samples to show very visually the typical behaviour of each concrete. In general, the graphs show that the R/PET-FRC specimens had a scarce reduction on the ultimate compressive strength than control samples, but the incorporation of the PET fibres generated in the concrete an augment on the compressive toughness. 

**Figure 6:**
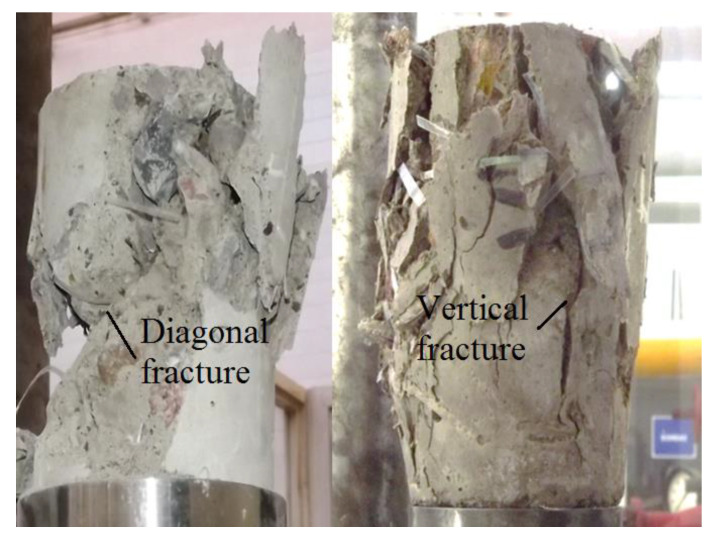
Compressive failures on R/PET-FRC samples.

The ultimate compressive strength reduction is related to the effect of the encapsulated air and porosity due to the incorporation of the fibres into the concrete, and its low adherence of the straight PET recycled fibres with a concrete matrix [19,38,39,40]. Figure 6 shows the typical fracture patterns observed on the R/PET-FRC samples, which have diagonal and vertical fractures. These failures produced that the recycled fibres are under a combination of stress mode.

#### 4.2.1. Ultimate compressive strength

**Figure 7:**
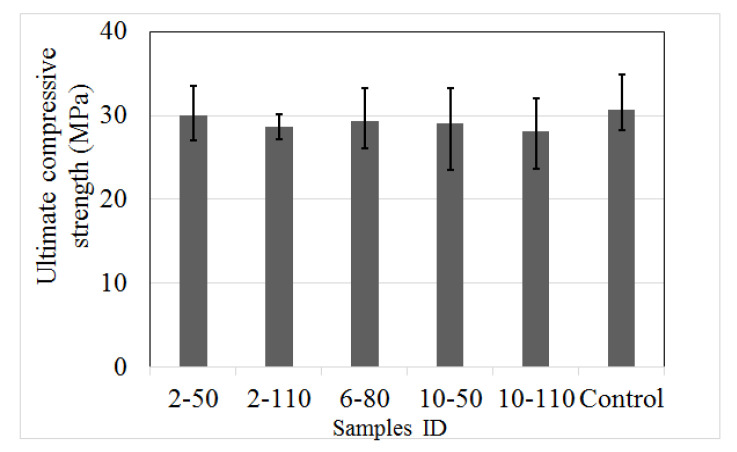
Ultimate compressive strength of control samples and concrete with recycled PET fibres.

Figure 7 displays the ultimate compressive strength (*f´_c_*), calculated with Equation (1). The results show that control samples have a mean of 30.8 MPa, which is 6% higher than the average of all the R/PET-FRC specimens. On the other hand, the variability of the results is another factor related to the recycled reinforced elements, samples with the highest PET fibre dose presented superior variability than those with low reinforced elements or those without fibres. Also, the reduction in the *f´_c_* is related to the fibre AR, samples with the longest PET fibre had lower performance than those with short dimensions.

(1) $$f{´}_{c}\;=\;\frac{4000F_{c}}{\pi D^{2}}$$

where:

*f´_c_*: ultimate compressive strength
*F_c_*: ultimate compressive load
*D*: sample diameter

#### 4.2.2. Modulus of Elasticity

**Figure 8:**
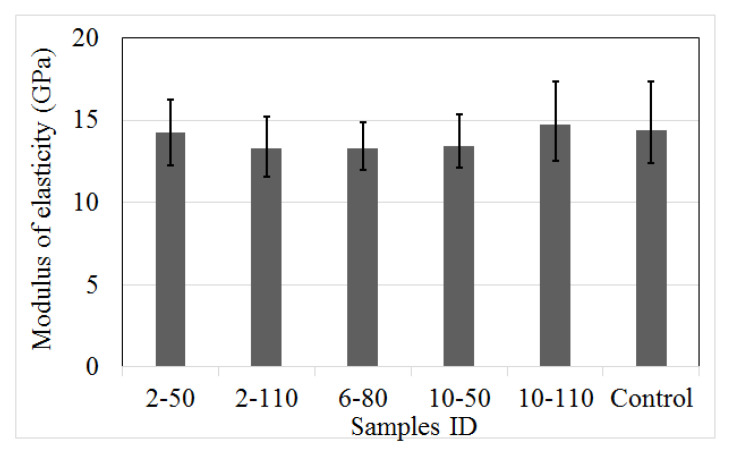
Modulus of elasticity of control samples and concrete with recycled PET fibres.

Figure 8 illustrates the results of the modulus of elasticity (E), determined according to the norm NMX-C-128-ONNCCE [41]. Equation (2) shows the relation used to calculate E. The results indicate that the change of the recycled PET fibre dose and aspect ratio generates a scarce difference of 10.1%. This effect produced that some R/PET-FRC concretes have higher rigidity (3%) and others a reduction of 1% to 8%, respecting to control samples. Alfabdawi et al. [42] and Kim et al. [9] observed a small variation in the modulus of elasticity of R/PET-FRC due to the change of the reinforced dose, but similar behaviour than control samples. In general, the results prove that the incorporation of recycled PET fibre into concrete has a low effect on the modulus of elasticity of the FRC matrix, with similar variability in the response between control samples and those with recycled fibres.

(2) $$E\;=\;\frac{f{´}_{c}^{40\%}-f{´}_{c}^{0.00005}}{\epsilon^{40\%}-\varepsilon^{0.00005}}$$

where:

*E*: modulus of elasticity
*f´_c_*^40%^: compressive strength corresponding to 40% of the maximum value
*f´_c_*^0.00005^: compressive strength corresponding to 0.00005 of the strain registered
*ε*^40%^: strain corresponding to *f´_c_*^40%^
*ε*^0.00005^: strain corresponding to *f´_c_*^0.00005^

#### 4.2.3. Compressive Toughness

**Figure 9:**
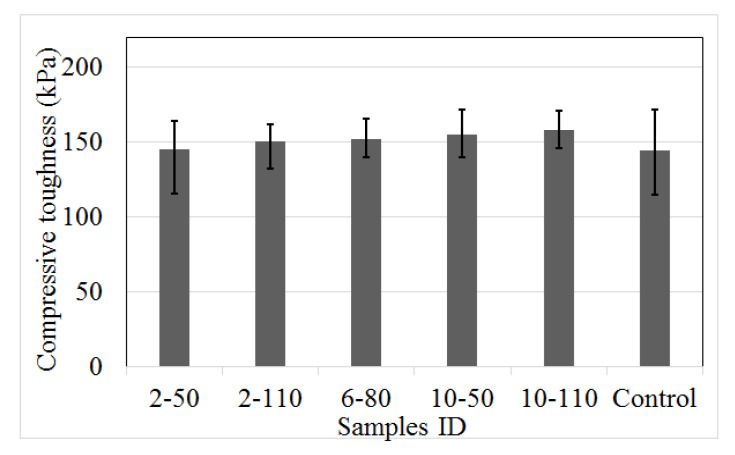
Compressive toughness of control samples and concrete with recycled PET fibres.

Figure 9 shows the compressive toughness (*T´_c_*) of the recycled PET fibre reinforced concrete and control samples. *T´_c_* represents the residual strength of a concrete matrix, calculated through the capacity of energy based on the compressive strength-strain graph (see Figure 5). Equation (3) indicates the relation used to calculate *T´_c_*. The mean illustrates an augment of the *T´_c_* on R/PET-FRC concretes respecting to control samples, with a difference from 1% to 9%. This finding proves that the incorporation of recycled PET fibres in the concrete generates a scarce augment on the compressive energy capacity; property related to the combination of stress mode (flexion, shear, compression, and tension). According to Kim et al. [9] and Vázquez [43], the most unfavourable cases for the PET material are when the PET is under compression, flexion, or shear load; due to the low rigidity and low shear strength of the recycled PET fibres. On the other hand, the data show lower variability on the T´c of the R/PET-FRC samples than on control samples, representing a considerable property due to the recycled reinforced elements. Also, the best compressive toughness was on FRC samples with high fibre dose and AR.

(3) $$T{´}_{c}=\int_{0}^{3\varepsilon_{1}}\sigma_{c}d\varepsilon$$

where:

*T´_c_*: compressive toughness
*σ_c_:* compressive strength
*ε*: strain
*ε*_1_: strain corresponding to the maximum compressive strength

### 4.3. Splitting Tensile Behaviour

**Figure 10:**
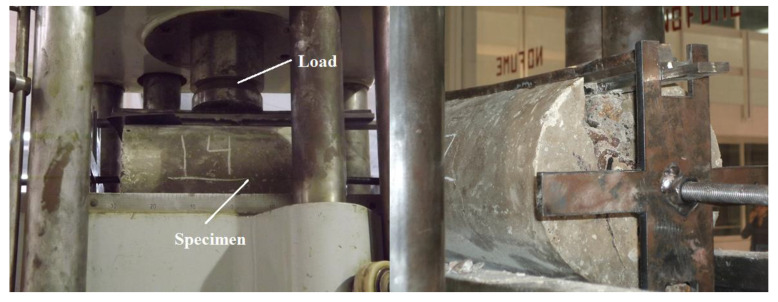
Splitting tensile test.

**Figure 11:**
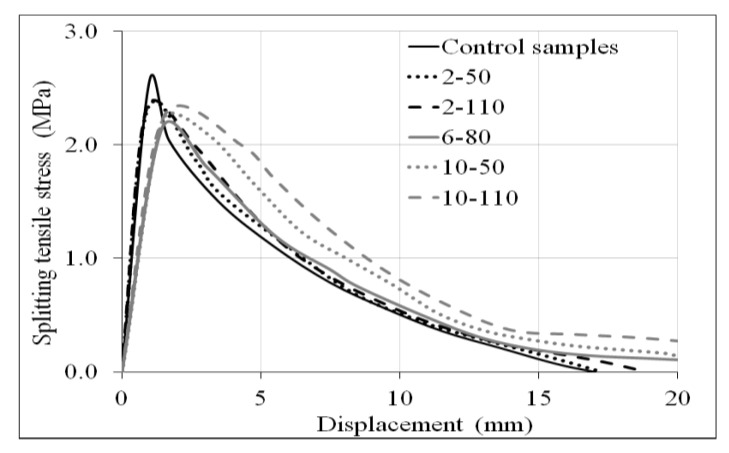
Splitting tensile stress-displacement curves of control samples and concrete with recycled PET fibres.

Samples of 150 × 300 mm (diameter and length, respectively), according to ASTM C496 [44], were tested. Figure 10 shows the Jig used for the splitting tensile test, and Figure 11 displays the splitting tensile stress-displacement curves of control samples and R/PET-FRC concretes.

In general, the curves demonstrate that the control samples have superior ultimate splitting tensile stress than those with recycled PET fibres, control samples reported a capacity of 2.6 MPa, and the mean of the R/PET-FRC samples 2.3 MPa. On the other hand, the recycled reinforced elements generated more ductility to the concrete, with a higher area under the curve on R/PET-FRC samples. The splitting tensile behaviour was studied with two parameters: the ultimate splitting tensile strength (*f´_t_*) and the tensional toughness (*T´_t_*).

#### 4.3.1. Ultimate Splitting Tensile Strength

**Figure 12:**
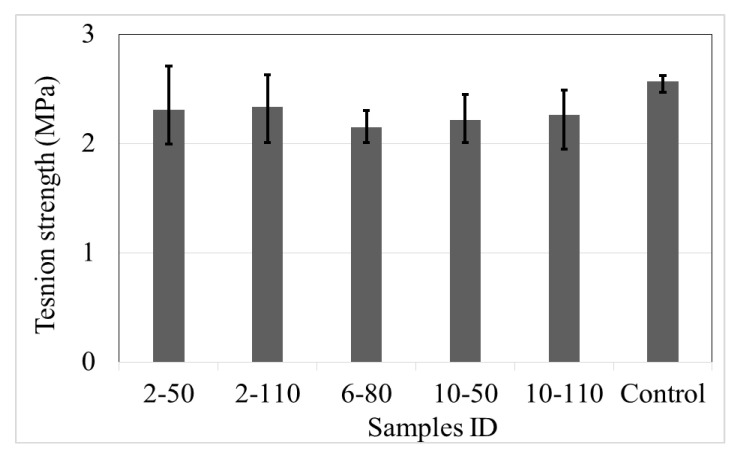
Splitting tensile strength of control samples and concrete with recycled PET fibres.

Figure 12 displays the results of the ultimate splitting tensile strength (*f´_t_*), calculated with Equation (4). The mean results indicate that the incorporation of recycled PET fibres into the concrete produces a reduction from 9% to 16% (with respect to control samples). However, Mostafa et al. [23] reported an augment in the *f´_t_* of recycled PET fibre reinforced concrete to control samples, with a maximum difference of 19%. 

In general, the data prove that the incorporation of fibres into concrete generates a limited positive or negative effect in the ultimate splitting tensile strength, with scarce consequence due to the change of fibre dose and aspect ratio (maximum of 8%). Nevertheless, the data show an important augment of the variability between the limits of the response; control samples had a difference of 0.15 MPa, while the R/PET-FRC concretes had an average of 0.52 MPa.

(4) $$f{´}_{t}\;=\;\frac{2P}{\pi ld}$$

where:

*f´_t_*: ultimate splitting tensile strength
*P*: ultimate splitting tensile load applied
*l*: sample length
*d*: sample diameter

#### 4.3.2. Tensional Toughness

**Figure 13:**
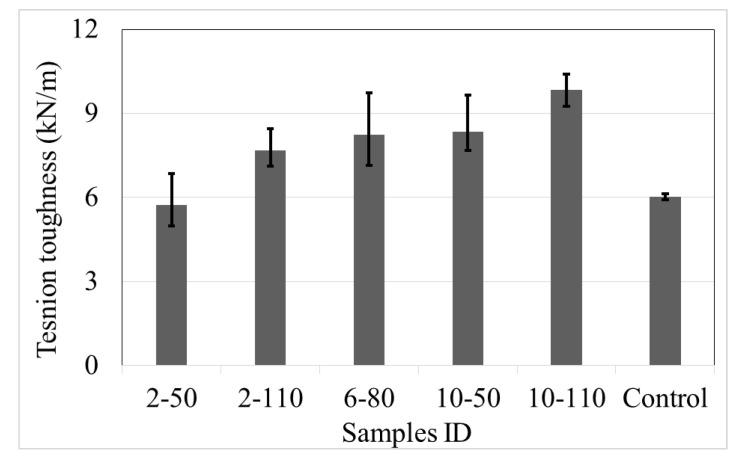
Tensional toughness of control samples and concrete with recycled PET fibres.

Figure 13 displays the results of the tensional toughness (*T´_t_*), calculated with Equation (5). *T´_t_* represents the area under the curve splitting tensile strength–deformation and denotes the energy absorption after the first crack is deemed to have occurred in a concrete matrix. The results show that the *T´_t_* depends on the recycled PET fibre parameters. High proportions of dose and aspect ratio prove the best performance; in comparison with control samples, 10-110 increases in 39%, while 2-15 reduces in 5%. Another effect of the recycled PET fibres into concrete was the variability in the responses, which was superior to control samples (0.2 kN/m on control samples and an average of 1.8 kN/m on R/PET-FRC). Despite the variability, 88% of the specimens with recycled PET fibres have superior performance to control samples. In general, the data demonstrate a considerable contribution of the recycled PET fibre in the tensional toughness of a concrete matrix, which depends on the parameters of the fibres.

(5) $$T{´}_{t}\;=\;\int_{0}^{3\delta_{1}}\sigma_{t}d\delta_{t}$$

where:

*T´_t_*: tensional toughness
*σ_t_*: splitting tensile strength
*δ_t_*: splitting tensile deformation
*δ*_1_: deformation corresponding to the maximum splitting tensile strength

### 4.4. Flexural Behaviour

**Figure 14:**
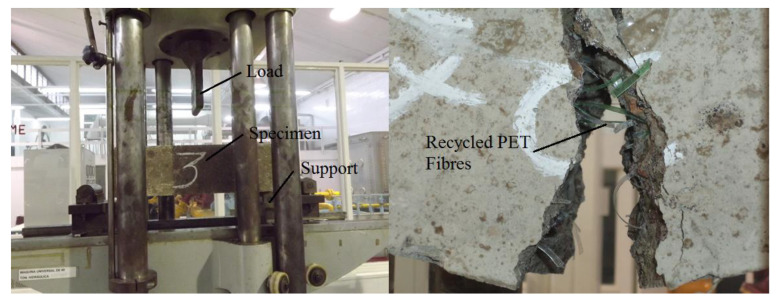
Flexural test.

**Figure 15:**
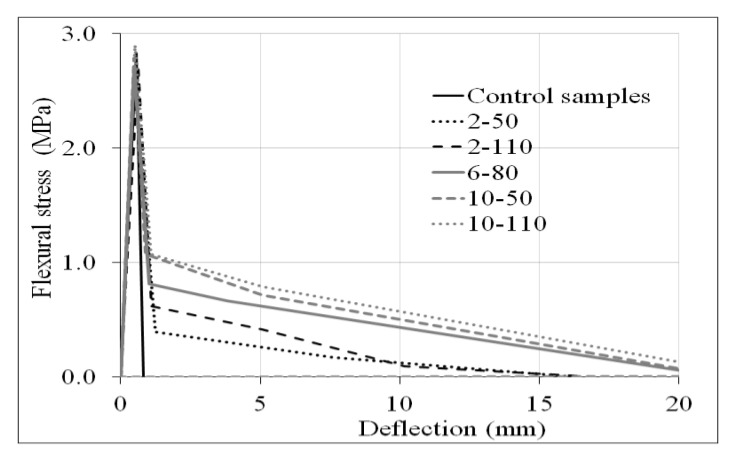
Flexural stress–deflection curves of control samples and concrete samples with recycled PET fibres.

Figure 14 and Figure 15 illustrate the flexural test and the average flexural stress–deflection curves of control samples and R/PET-FRC concretes, respectively. As for the compressive test, the average results are provided to show the typical behaviour of each concrete. The specimens had dimensions of 150 × 150 × 500 mm and were tested according to ASTM C78, adapted to the center-point loading arrangement [45,46]. 

The midspan deflection was measured using a plunger dial indicator located on the testing machine following the same procedure as in the compressive test. The graphs show the ductility capacity that the concrete acquires with the incorporation of the PET fibres; while the control samples present brittle failure after the peak flexural stress, the concrete with recycled PET fibres provide residual strength. Flexural behaviour was evaluated through the ultimate flexural strength and the equivalent flexural strength ratio.

#### 4.4.1. Ultimate Flexural Strength

**Figure 16:**
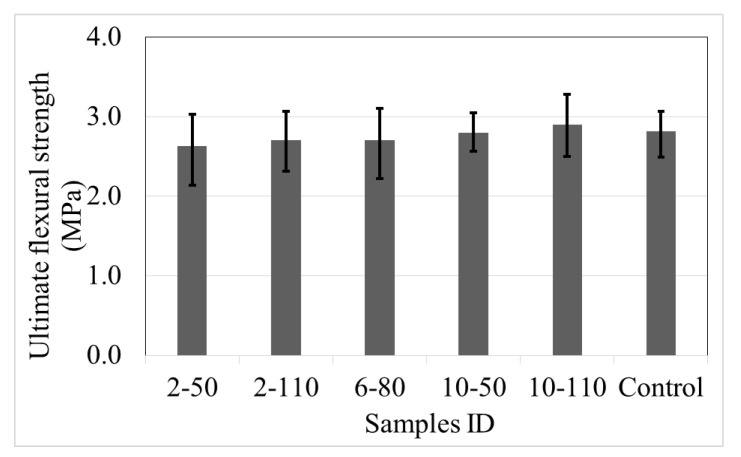
Ultimate flexural strength of control samples and concrete with recycled PET fibres.

Figure 16 exhibits the ultimate flexural strength (*f´_f_*) of control samples and those with recycled PET fibres, calculated with Equation (6). The average data indicate that the recycled PET fibre reinforced concretes have *f´_f_* comparable to the control samples, with a difference of 7%. Other studies reported a similar strength Borg et al. [6] point out that the concrete with recycled PET fibres reaches an increment of 15%, while Ochi et al. [14] express a reduction of 3%. On the other hand, the change of the recycled PET fibre dose or aspect ratio into concrete proves a limited influence on the *f´_f_*, with similar variability between its limits.

(6) $$f{´}_{f}\;=\;\frac{P_{f}L_{f}}{b_{f}d_{f}{}^{2}}$$

where:

*f´_f_*: ultimate flexural strength
*P_f_*: flexural load peak
*L_f_*: span length
*b_f_*: average width
*d_f_*: average depth

#### 4.4.2. Equivalent Flexural Strength Ratio

**Figure 17:**
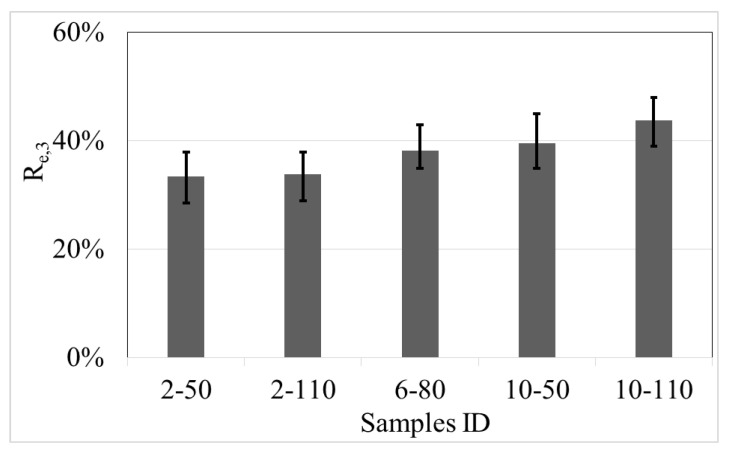
Equivalent flexural strength ratio of concrete with recycled PET fibres.

Figure 17 indicates the equivalent flexural strength ratio (*R_e,_*_3_) of recycled PET fibre reinforced concrete. *R_e,_*_3_ of the control samples is null because it does not contain reinforced elements. The mean data shows that *R_e,_*_3_ of recycled PET fibre reinforced concrete is between 33% and 44%, samples with a high proportion of fibre dose and aspect ratio have the best residual strength. The Concrete Society [32] recommends a minimum residual equivalent flexural strength ratio of 30% for industrial application, which is a criterion fulfilled by the fibre-reinforced concrete in all the combinations. Also, the data show that the variability of the responses was similar between the fibre–concrete samples. Equation (7) expresses the relation used to calculate *R_e,_*_3_, according to JSCE [47].

(7) $$R_{e,3}\;=\;\frac{150\int_{0}^{3\delta_{f}}F\delta }{P_{f}L_{f}}$$

where:

*R_e,_*_3_: flexural strength ratio
*P_f_*: flexural load peak
*L_f_*: span length
*δ*: deflection
*δ_f_*: *P_f_* deflection

### 4.5. Volumetric Weight

**Figure 18:**
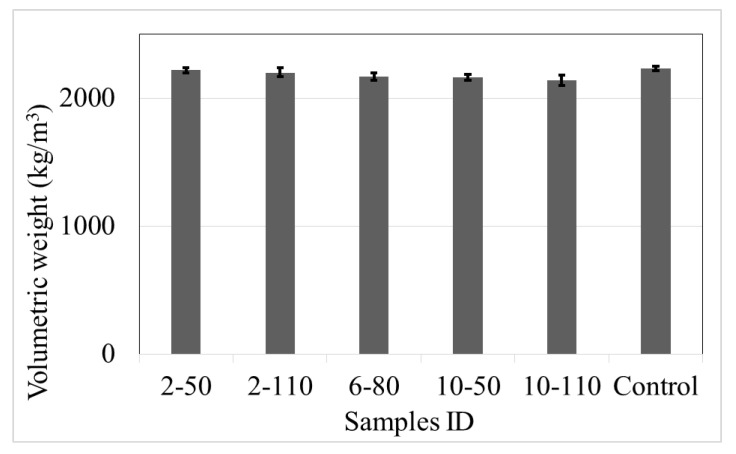
Volumetric weight of control samples and concrete with recycled PET fibres.

Figure 18 indicates the volumetric weight (V_W_) of the control samples and R/PET-FRC concretes, measured according to ASTM C78 [46]. 

The results show that the incorporation of recycled PET fibres in the concrete generates an irrelevant reduction in the volumetric weight, with a difference of 1% to 4% in comparison with control samples. Pereira and Castro [48] informed similar behaviour on concrete with recycled PET fibres, with a difference of 5%. Also, the variability of the volumetric weight between samples was low, with a proportion similar to control samples.

### 4.6. Fibres Per Square Meter

**Figure 19:**
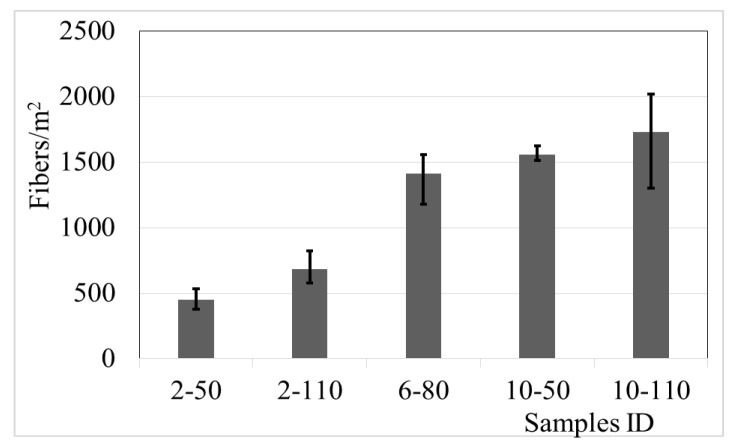
Number of fibres per square meter of concrete with recycled PET fibres.

Broken samples, after the flexural test, were used to count the number of fibres on the cracking section. Figure 19 shows the results of the fibre per square meter (*N_f_*). 

The data exhibit that the *N_f_* increases with the proportion of recycled PET fibres dose and aspect ratio, with a considerable difference (74%) between the samples 2-50 and 10-110 (minimum and maximum, respectively); the effect of the augment of the *N_f_* with the aspect ratios is attributed to the property of a better arrangement of the long fibres into the concrete.

## 5. Statistical Analysis

Table 2 indicates the DOE of recycled PET reinforced concretes, considering the five sample replicates and the randomization. The information in Table 2 was fed in the Minitab software to study if the change of the recycled PET fibres dose or aspect ratio had a statistical impact on the responses. Minitab is a statistical package for the analysis, estimation, and presentation of data, where statistical procedures can be applied for the evaluation of results. 

In this study, the analysis considers the answers (slump, ultimate compressive strength, compressive toughness, modulus of elasticity, ultimate tensile strength, tensional toughness, ultimate flexural strength, equivalent flexural strength ratio, volumetric weight, and the number of fibres). Additionally, regression and optimization treatments are the statistical methodologies that complemented the analysis.

AR is the recycled fibre aspect ratio, *f´_c_* is the ultimate compressive strength, *E* is the modulus of elasticity, *T´_c_* is the compressive toughness, *f´_t_* is the ultimate tensile strength, *T´_t_* is the tensional toughness, *f´_f_* is the ultimate flexural strength, *R_e,_*_3_ is the equivalent flexural strength ratio, *V_W_* is the volumetric weight, and *N_f_* is the fibres per square meter.

### 5.1. Factor Analysis

Factor analysis considers the effect of the factors in the responses based on their variability, relative response, and confidence. These parameters are evaluated through the *F*-value, which is desirable to be equal or less than 0.05, meaning a significance level of 5%, with a confidence value equal to 95% [29,49]. The statistical evaluation criteria and the number of samples proposed are consistent with other investigations related to the mechanical study of concrete [28,29,30].

**Figure 20:**
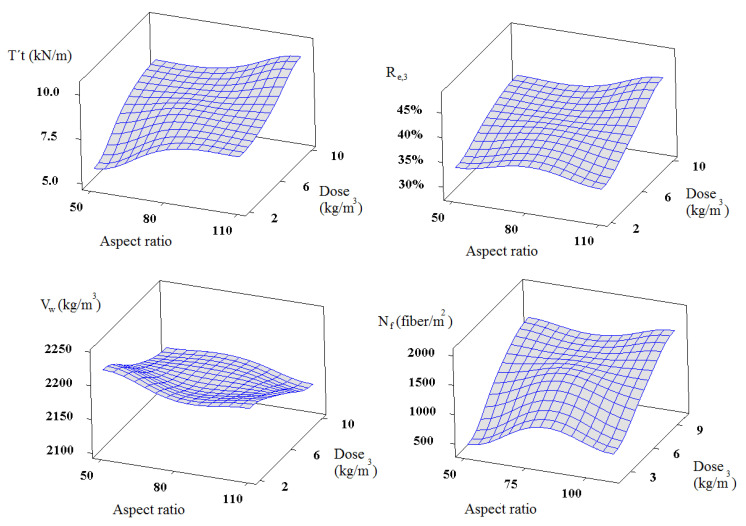
Surface graphs of fibre reinforced concrete (FRC) with recycled PET fibre.

In this research, the factors are the recycled PET fibre dose and aspect ratio, considering the individual and combined effect of the parameters. The results indicate that the factor fibre dose had statistical relation with the *T´_t_*, *R_e,_*_3_, *V_W_*, and N_f_. Also, the data show that the augment of the fibre dose produces an increment of the tensional toughness, equivalent flexural strength ratio, and the number of fibres, and a reduction of the volumetric weight. On the other hand, the fibre aspect ratio only demonstrates a statistical effect on the tensional toughness (the best response was with the longest fibres). Figure 20 shows the effect of the recycled PET fibre dose and aspect ratio on those responses that fulfil the statistical criterion.

### 5.2. Regression Analysis

Factor analysis demonstrated that the responses of the concrete with recycled PET fibres have high variability, even though some of them prove statistical significance; thus, regression analysis, to those responses with a value of confidence equal or superior to 95%, was applied. Equations (8)–(12) show the relations that explain the parameters based on recycled PET fibres dose or aspect ratio.

(8) $$T{´}_{t}\;=\;6.17+0.299Dose$$

(9) $$R_{e,3}\;=\;0.317+0.0101Dose$$

(10) $$V_{w}\;=\;2221-7.21Dose$$

(11) $$N_{f}\;=\;361+135Dose$$

(12) $$T{´}_{t}\;=\;5.67+0.0287AR$$

where

*T´_t_*: tensional toughness
*R_e,_*_3_: equivalent flexural strength ratio
*V_W_*: volumetric weight
*N_f_*: number of fibres on the cracking section of a flexural sample
Dose: RF dose
AR: RF aspect ratio

### 5.3. Optimization Analysis

**Figure 21:**
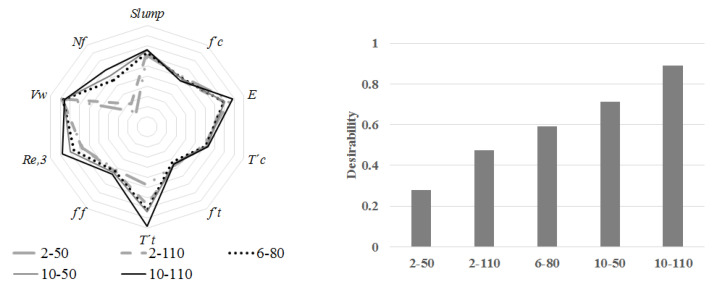
Comparison on the responses of recycled PET fibre reinforced concrete with different doses and aspect ratios and its statistical desirability.

The optimization analysis, in Minitab, selects several starting points to search for the optimal factors configuration (fibre dose and aspect ratio). The response optimizer grades the desirability with a scale of 0 to 1 (1 means the perfect solution). This study aims to find the minimization of the volumetric weight and the maximization of the remaining responses. The results indicate that concretes with high recycled PET fibre dose have the best performance. Specimens 10-110, 10-50, and 6-80 demonstrate a global desirability of 0.89, 0.71, and 0.59, respectively. On the other hand, samples 2-50 and 2-110 have values of 0.27 and 0.47. Figure 21 depicts the comparison between the relations studied. The samples 10-110 prove the best workability, rigidity, compressive toughness, tensional toughness, ultimate flexural strength, equivalent flexural strength ratio, fibres per square meter, and volumetric weight.

## 6. Comparative Performance of Optimal R/PET-FRC

This section makes a comparison of the optimal R/PET-FRC concrete (samples 10-110) respecting FRC with commercial steel and synthetic fibres. The study takes different researchers as a reference. Considering that the R/PET-FRC mix design could be different from other investigations, the capacity analysis is referenced to control samples (without fibres) to make an equivalent quotation. The results indicated the following:

Workability: the results indicated a similar reduction on the optimal concrete matrix with PET recycled fibres and those with steel elements. Recycled PET fibre reinforced concrete 10-110 had lower workability (19%) with respect to control samples, while the Ragalwar et al. [50] and Zemir et al. [51] indicated a decrease from 9% to 15% of SFRC with 1% of steel fibres; this percentage is equivalent to the fibre dose used in this samples 10-110.

Ultimate compressive strength: similar to SFRC, the effect of the optimal recycled PET fibres into concrete is negligible. Vairagade and Kene [52], and Ragalwar et al. [50] reported an augment of 6% to 10% in the response of SFRC samples with a fibre dose of 0.5 to 1%, referenced to control samples. Moreover, the optimal PET recycled fibre reinforced concrete was registered a reduction of 9%.

Modulus of elasticity: the response of the optimal R/PET-FRC had a scarce augment compared with control samples; the difference is similar to those found on SFRC and FRC with polypropylene reinforced elements [38].

Compressive toughness: The compressive toughness of the optimal R/PET-FRC is similar to SFRC. The post cracking compressive capacity of PET recycled fibre reinforced concrete 10-110 has a scarce augment of 9%, with respect to the control samples; while the incorporation of steel fibre dose of 3% proved an incrementation of 12% in SFRC [38].

Ultimate splitting tensile strength: the optimal PET recycled fibre reinforced concrete demonstrated a lower strength than SFRC. Vairagade and Kene [52] reported an augment of 14% on the ultimate splitting tensile strength on SFRC with a dose of 0.5%; while the samples 10-110 had a reduction of 12% compared to control samples.

Tensional toughness: the optimal R/PET-FRC demonstrated a superior tensional toughness with respect to control samples versus those found on FRC with polypropylene fibres [53,54,55,56,57]. According to the data reported by Abdulwahab et al. [47], the tensional toughness of the FRC with polypropylene fibres had an augment of 33% with respect to control samples, while samples 10-110 demonstrated an augment of 39%. Ultimate flexural strength: the response between the FRC with polypropylene fibres and those with the optimal PET recycled fibres demonstrates similar performance as the control samples. The maximum difference respecting concrete without fibres was 4% [38]. Equivalent flexural strength ratio: the optimal R/PET-FRC has a residual strength of 44%, similar to those reported on concrete with polypropylene fibres with equivalent fibre dose [38].

## 7. Conclusions and Future Scope

The purpose of this study is to find the best relation of fibre reinforcement of the concrete with recycled PET fibres under different tests. The experimental trials consider workability, compressive, splitting tensile, and flexural behaviour. Full factorial analysis was used to study statistically ten responses, controlled by the variation of the recycled fibre dose and aspect ratio. The results demonstrated that the incorporation of recycled PET fibres into concrete generates residual strength capacity to the concrete, with a scarce effect on its volumetric weight, and ultimate flexural and compressive strength. The statistical analysis of the factors indicated that the recycled fibre dose has more influence on the responses than the fibre aspect ratio. The fibre dose was related to the tensional toughness, equivalent flexural strength ratio, volumetric weight, and the number of fibres while the aspect ratio has a statistical impact on the tensional toughness. According to the optimization study, the optimal R/PET-FRC is the concrete with the highest fibres dose and aspect ratio. This relation proved the best workability, modulus of elasticity, compressive toughness, tensional toughness, ultimate flexural strength, and equivalent flexural strength ratio. Furthermore, the levels of the compressive toughness, flexural toughness, and tensional toughness of the optimal R/PET-FRC are similar to those of samples with commercial fibres in equivalent dose proportions. The study strengthens the use of concrete with recycled PET fibres by understanding its behaviour, which could help to understand the mechanical strength of FRC structures with recycled PET fibres.

## Figures and Tables

**Table 1:** Mix proportions.

	R/PET-FRC			Concrete Mix Proportions (kg/m^3^)			
ID	Dose (kg/m^3^)	Longitude (mm)	Aspect Ratio	Cement	Natural Sand	Gravel (20 mm)	W/C Ratio
Control	0	0	0	383	672	1100	0.6
2-50	2	53.5	50	383	672	1100	0.6
2-110	2	53.5	110	383	672	1100	0.6
6-80	6	85.6	80	383	672	1100	0.6
10-50	10	117.8	50	383	672	1100	0.6
10-110	10	117.8	110	383	672	1100	0.6

**Table 2:** Results of concrete with recycled PET fibres in different doses and aspect ratios.

Test No.	Dose	AR	Label	Slump	*f´c*	*E*	*T´_c_*	*f´_t_*	*T´_t_*	*f´_f_*	*R_e,_*_3_	*V_W_*	*N_f_*
				mm	MPa	GPa	kPa	MPa	kN/m	MPa	%	kg/m^3^	Fibre/m^2^
3	2	50	2-50	360	29.2	13.6	164.3	2.2	6.6	3.0	29%	2236	378
6	2	50	2-50	385	33.5	13.9	155.9	2.7	6.8	2.4	36%	2196	444
13	2	50	2-50	308	27.0	15.1	115.5	2.0	5.2	2.1	33%	2229	533
17	2	50	2-50	356	28.2	16.3	140.2	2.5	5.0	2.8	38%	2210	480
22	2	50	2-50	346	32.2	12.3	149.7	2.1	5.0	2.8	32%	2230	423
1	10	50	10-50	400	33.2	15.4	171.8	2.2	8.5	3.0	45%	2150	1511
4	10	50	10-50	310	26.9	13.7	140.3	2.5	9.6	2.6	35%	2183	1556
7	10	50	10-50	348	23.5	13.3	163.5	2.0	7.7	2.7	41%	2141	1622
10	10	50	10-50	420	32.6	12.6	145.6	2.4	8.1	3.1	37%	2160	1580
18	10	50	10-50	410	29.3	12.1	155.1	2.1	7.8	2.7	40%	2170	1530
5	6	80	6-80	324	26.1	12.4	165.7	2.1	8.7	2.7	37%	2199	1178
9	6	80	6-80	335	32.4	14.2	150.2	2.2	9.7	2.2	36%	2146	1511
12	6	80	6-80	349	26.6	12.0	149.6	2.1	7.1	3.1	43%	2141	1556
16	6	80	6-80	400	33.2	13.1	154.6	2.3	7.6	2.5	35%	2185	1380
23	6	80	6-80	420	28.6	14.9	140.1	2.0	8.0	3.0	40%	2180	1440
8	2	110	2-110	430	29.0	13.1	157.7	2.3	7.1	2.5	29%	2178	578
14	2	110	2-110	366	29.5	13.2	162.3	2.6	7.6	2.3	33%	2198	667
19	2	110	2-110	317	27.6	13.4	132.3	2.6	8.4	3.1	36%	2200	822
24	2	110	2-110	381	30.1	15.2	153.2	2.1	7.2	2.8	38%	2170	701
25	2	110	2-110	361	27.1	11.6	147.5	2.0	8.0	2.9	33%	2240	660
2	10	110	10-110	402	31.9	13.5	171.1	2.5	10.4	3.3	43%	2126	2000
11	10	110	10-110	360	27.6	15.3	159.8	2.3	9.4	2.5	43%	2169	1300
15	10	110	10-110	373	23.7	12.5	148.3	2.0	9.2	2.7	39%	2101	2022
20	10	110	10-110	392	25.2	17.4	146.2	2.2	10.0	3.0	46%	2130	1600
21	10	110	10-110	372	32.1	15.2	163.9	2.31	10.13	3.1	48%	2180	1725

## Data Availability

Data available in a publicly accessible repository.
